# Perceptions and Referral Trends into Phase I Oncology Trials: Results of a Clinical Survey

**DOI:** 10.1155/2011/861401

**Published:** 2011-06-27

**Authors:** Andre Brunetto, David Olmos, Hendrik-Tobias Arkenau, Daniel Tan, Timomy Yap, Johann de Bono, Jorge Barriuso, Stan Kaye

**Affiliations:** ^1^The Royal Marsden Hospital, Surrey SM2 5PT, UK; ^2^Sarah Cannon Research UK, London W1G 6AD, UK

## Abstract

*Introduction*. A survey was sent to referring oncologists (ROs) to explore the reasons behind their referral patterns and perceptions of Phase I studies before and after being provided with outcome data from advanced colorectal cancer (ACRC) patients who participated in Phase I trials at the Royal Marsden Hospital (RMH). *Results*. The response rate was 32/50 (64%). The most common reason for referral was exhaustion of standard treatments (31%), and the main reason for referring to the RMH was proximity to patients (28%). The most frequent clinical parameter assessed prior to referral was performance status (93%). ROs spent a median of 15 min (range: 5–45 min) discussing general aspects of Phase I trials. In the second part of the questionnaire, after reviewing clinical outcome data of ACRC patients who participated in Phase I trials, 47% would change their approach, specifically, spend more time to discuss risks and benefits of Phase I trials (9%), consider prognostic factors before referral (13%), and increase the number of referrals (25%). *Conclusion*. This is the first report focusing on communication between ROs and a specialist Phase I unit. Outcome reporting can improve communication with ROs and importantly has the potential for better patient selection considered for Phase I oncology trials.

## 1. Introduction

The clinical outcome of Phase I trials is usually descriptive due to small patient numbers. Still, there is evidence that a significant number of patients with ACRC derive clinical benefit, at least in the form of meaningful prolonged disease stabilisation [1]. Moreover, patient selection based on clinical parameters such as albumin, LDH, and number of metastatic sites may be utilised to identify patients who may benefit from Phase I study treatment [2]. 

The motivation for patients to participate in Phase I trials is broad, ranging from personal benefit to pure altruism [3]. Unlike their treating oncologists, patients generally have high expectations with regards to clinical benefit and are even willing to accept greater drug-related toxicities from experimental therapy [4–6]. In this context patients rely heavily on the opinion and expertise of their oncologists to balance the risks and benefits of Phase I trial treatment. Yet, most general oncologists may often have limited experience with Phase I trials of modern agents. It is therefore important to gain a better understanding of referral patterns of ROs as well as their perceptions of Phase I clinical trials. In this study a structured questionnaire was sent to all oncologists who referred patients to the RMH for Phase I trials. The main goal of the study was twofold: (a) improve our understanding of the referral patterns of oncologists and (b) investigate their general perception of experimental therapy and the associated prognosis. Finally, we also aimed at obtaining feedback on clinical outcome data for ACRC patients who participated in Phase I trials and were part of a previous outcome analysis. Briefly, this analysis identified that a considerable amount of patients had clinical benefit, as measured in prolonged stable disease (SD) [1]. Patients with SD had generally a better RMH prognostic score and achieved an OS of 40.6 weeks compared to 17.4 weeks for patients with disease progression.

## 2. Methods

This study was carried out at the Royal Marsden Hospital, Surrey, UK, and was reviewed and approved by the institution's audit committee. 

**Figure 1:**
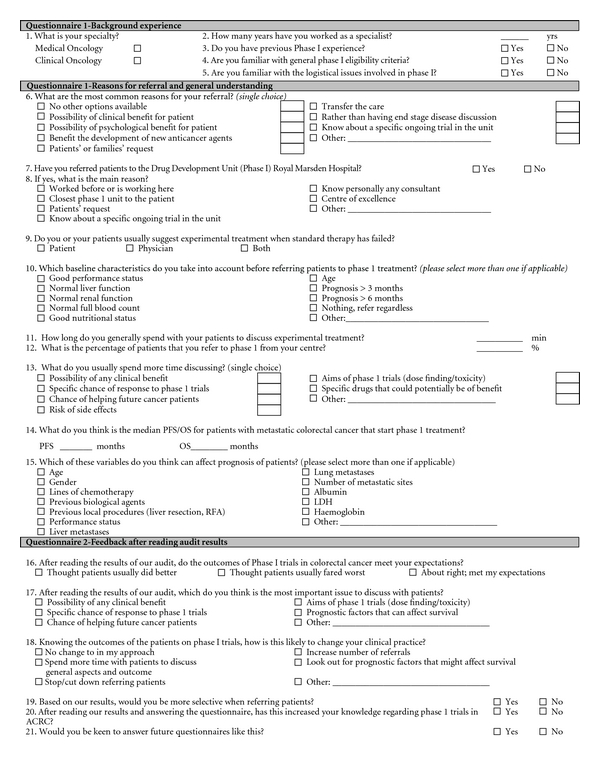


Two questionnaires were sent together (Figure 1): the first was designed to explore the perceptions of oncologists about Phase I clinical trials. Questionnaire 1 (questions: 1–5) included questions on the background of the referring physicians, their clinical experience and familiarity with general trial concepts, as well as on logistical issues involved in Phase I trials. In addition, Questionnaire 1 (questions: 6–15) evaluated the most common reasons for referral to Phase I clinical trials, reasons for referral to the RMH, baseline patient characteristics that were taken into account before referring them for Phase I treatment, estimated progression-free survival (PFS) and overall survival (OS) for patients with ACRC participating in Phase I trials, and variables that could affect the patients' prognosis. The first questionnaire had to be filled prior to reviewing the clinical Phase I outcome results for ACRC patients (summary of results shown in Table 1). Following this participants were invited to complete questionnaire 2.

The second questionnaire (questions 16–21) assessed if a better knowledge regarding clinical Phase I trial outcome for ACRC patients would affect referral trends of ROs to Phase I trials and whether this would impact on their clinical practice. Overall, a total of 21 questions were included in both questionnaires, some being single and others multiple choice questions. All four pages (invitation letter, Questionnaire 1, clinical outcome data, and Questionnaire 2) were sent in the same envelope in this exact order with an additional preposted envelope enclosed. The responses to the Questionnaires were kept confidential and contained no identifying codes or tags, making it anonymous. For this reason, a general reminder was sent in 3 weeks time. At the end of Questionnaire 2 there was space for any additional comments one should wish to make. Participants were invited to give their names if they were interested in getting feedback on the results of the survey. 

All statistical analyses were performed using the SPSS Software (Version 12.0; SPSS, Chicago, Ill, USA). Descriptive statistics on survey questionnaire are presented, and total percentages are shown.

## 3. Results

### 3.1. Questionnaire 1

Thirty-two out of 50 questionnaires (64%) were returned from medical and clinical oncologists (16 each). The median clinical experience in the field of oncology was 10 yrs (range: 2–25). Sixty-six percent of the oncologists had previous Phase I experience, and more than 80% were familiar with logistics and general eligibility criteria for Phase I trials.

The most common reason for referring patients for participation in Phase I clinical trials was lack of treatment options and benefit development of new agents, see Table 2. The discussion of Phase I trials was found to be initiated by ROs in more than 50% of the cases and only in less than 10% of the cases by the patient. The reasons for referral specifically to the RMH Drug Development Unit were found to be the proximity to the patients' home (28%), the awareness of specific trials ongoing at the RMH unit (22%), considering RMH a centre of excellence (22%), knowing a consultant at the unit personally (13%), previously working at RMH (9%), and patients' request (3%). 

The most common baseline characteristics of patients that are taken into account before referral were performance status (93%), life expectancy greater than 3 months (59%), and normal renal function (56%) (see Table 3). The ability to travel as well as time involvement was additional factors which were added by the respondents and were not part of the original options of the questionnaire.

 ROs replied that they refer about 5% (range 0.5–20%) of their ACRC patients for consideration of Phase I trials and that they spend a median time of 15 min (range: 5–60 min) to discuss Phase I trials. The main points discussed with patients are the possibility of clinical benefit and the aims of Phase I trials (see Table 4). The oncologists estimated the median PFS and OS of ACRC patients undergoing Phase I trials to be 8 and 24 weeks, respectively. The most prominent variables oncologists felt impact on clinical outcome were low albumin, poor PS, and number of metastatic sites (see Table 5).

### 3.2. Questionnaire 2

After completing Questionnaire 1 and having read the results of our clinical audit (resume in Table 1) ROs were asked to complete Questionnaire 2. Overall 25/32 (78%) of the ROs stated that the clinical outcome data for patients with ACRC in Phase I trials met their expectations. Six out of the 32 (19%) ROs estimated that patients would have a worse outcome and only 1 in 32 (3%) expected patients to have a better outcome if they were not included in the trial. Importantly, 25% of ROs felt that the clinical outcome results they just read will impact on their future patient selection. Moreover, the majority of oncologists (88%) replied that our questionnaire in combination with the outcome data increased their knowledge with regards to Phase I oncology trials, and 81% of ROs declared their interest in being involved in similarly structured surveys in the future. As a result of this survey, 9% of the oncologists would increase their time with patients to discuss Phase I trial aspects and outcome, 13% said they would include prognostic factors associated with survival before referring patients, while 25% are planning to increase their number of referrals.

## 4. Discussion

Patients who participate in Phase I trials have often high expectations of deriving personal clinical benefit despite the fact that benefit from Phase I trial participation is generally limited [2, 6, 8]. Given their short life expectancy and lack of realistic treatment options patients may be vulnerable. One of the paramount roles for oncologists in this setting is to advise patients in decision-making and also to balance potential risks and benefits of participating in Phase-I trials. Moreover, the major challenges of Phase I clinical trials are to select those patients who are suitable for trial participation, to ensure that use of resources is optimized, and that the burden to patients and their families is minimized. Clearly, in this situation it is crucial to have a close collaboration between the Phase I specialist, the referring physician, and the palliative care team in order to optimise the patient's benefit [7, 9, 10].

General eligibility criteria for Phase I trials include life expectancy greater than 3 months, and this is subject to clinical evaluation by treating physician and background experience. Prognostic tools can aid in patient selection and be incorporated to Phase I clinical trial protocols aiming to minimise the burden to patients by excluding those who are inappropriate for trial participation. In addition, palliative care programs have reduced costs, may be more convenient for patients, and are considered by many a major component of cancer care. Palliative care programs improve symptom control and the overall quality of life of patients suffering from progressive diseases and offer improved support for their families [7, 10, 11].

The ability of clinicians to predict survival accurately in terminally ill cancer patients historically has been unreliable, and even experienced physicians overestimate survival [12]. Therefore, there was a need for objective parameters to help predict outcome, and our institution developed a prognostic score that was prospectively validated and associated with poor outcome. Low albumin (<35 mg/dL), lactate dehydrogenase (LDH) above the upper limit of normal range and more than two sites of metastasis were independent negative prognostic factors defining a risk score able to identify a group (score 2-3) with poor prognosis [13]. In this context, we analysed the outcome of a large cohort of ACRC patients who participated in Phase I trials of targeted agents and confirmed that a high prognostic score was also associated with poor OS [1].

In our survey oncologists named several parameters associated with clinical outcome including performance status, albumin, LDH, and number of metastatic sites. Interestingly, these factors were also part of our recently developed prognostic score. Other factors such as previous chemotherapy lines and the presence of liver metastases, which are commonly regarded as negative prognosticators, were mentioned to a lesser extent. None of the ROs regarded gender to be associated with poor outcome, although our recent ACRC study showed a gender-related difference for survival. Gender disparities and prognosis of colorectal cancer have been described and may be associated with hormonal status playing an important role in the pathogenesis of colorectal cancer [14]. 

In estimating the PFS and OS for ACRC patients on Phase I trials, the respondents came close to the outcome data of the trial. The median estimated PFS and OS were found to be 8 and 24 weeks respectively, compared with the actual values of 8.6 and 29.1 weeks. Twenty-five per cent of ROs stated that the referral to Phase I trials was made in order to benefit the development of new compounds. A significant number of ROs having previous experience with early phase trials combined with the lack of exciting third-line options for colorectal cancer within the United Kingdom National Health System may have predisposed these numbers.

In the second part of the present survey, oncologists were asked to give feedback on whether our clinical outcome data may impact their future decision-making and subsequently result in increasing Phase I referrals. Interestingly, nearly 50% of ROs stated they will consider the Phase I prognostic score for patient selection in the future. Additionally, the respondents are aiming to increase the time they spend to discuss different aspects of Phase I trials with their patients and, as a direct result of the provided data, one quarter plan to increase the number of referrals to our centre. 

We are aware that this questionnaire may have limitations, nevertheless the results of this survey have helped us to better understand on what basis oncologists select patients for Phase I trials. Moreover, the active exchange of clinical outcome data with our referring centres could result in improving their understanding of Phase I trials and may further optimize patient selection. We trust that active engagement of ROs has the potential to improve patient care by minimizing the referral of poor prognosis patients.

## Figures and Tables

**Table 1:** Outcome results for Phase I trials for patients with advanced colorectal cancer (ACRC).

Overall survival (median)	29.1 weeks		Log-rank *P* = 0.029
Progression-free survival (median)	8.6 weeks		
OS RMH prognostic score 0-1	47.4 weeks	95% CI 41.3–53.6	
OS RMH prognostic score 2-3	19.4 weeks	95% CI 11.7–27.1	

Prognostic factors	Univariate (log-rank) *P* =	Multivariate (Cox regression) *P* =	

Age (<62 versus >62 years)	0.653	—	
Gender (male)	0.021	0.009	
Lung metastases	0.520	—	
Liver metastases	0.050	0.508	
Previous use of biological agents	0.661	—	
Previous local procedures	0.120	—	
RMH score	0.029	0.007	

Drugs Class	Number of trials	Number of patients	

HDAC/antisense/DNA repair	6	17	
Growth factor receptor inhibitor	5	11	
Antiangiogenesis	7	15	
Cell cycle/apoptosis	3	9	
Virus/vaccinia	1	3	
Various survival pathways	6	23	

RMH: Royal Marsden Hospital; CI: confidence interval; Hdac: histone deacetylase; Various survival pathways: including Akt/PI3 kinase/MTOR pathway.

**Table 2:** Reasons for referral to Phase I trials? (single choice).

(1) No other options available	31%
(2) Benefit development of new agents	25%
(3) Possibility of clinical benefit for patient	20%
(4) Patients' or families' request	10%
(5) Possibility psychological benefit for patient	8%
(6) Know about a specific ongoing trial	3%
(7) Rather than having end stage disease discussion	3%

**Table 3:** Which baseline characteristics do you take into account before referring patients to Phase I treatment? (multiple choice).

(1) Good performance status	93%
(2) Life expectancy longer than 3 months	59%
(3) Normal renal function	56%
(4) Normal liver function	50%
(5) Normal full blood count	40%
(6) Good nutritional status	37%
(7) Life expectancy longer than 6 months	28%
(8) Age	25%
(9) Normal albumin	3%
(10) Ability to travel to Phase I unit	3%

**Table 4:** Issues discussed with patients before referral to Phase I trials? (single choice).

(1) Aims of Phase I (dose finding and toxicity)	25%
(2) Possibility of any clinical benefit	23%
(3) Chance of helping future cancer patients	17%
(4) Specific chance of response to Phase I	15%
(5) Risk of side effects	14%
(6) Specific drugs that could potentially help	3%
(7) Others ( Travel required )	3%

**Table 5:** Which of these variables do you think affect prognosis of patients? (multiple choice).

(1) Albumin	97%
(2) Performance status	94%
(3) Number of metastatic sites	78%
(4) Lines of chemotherapy	62%
(5) Liver metastases	56%
(6) Lactate dehydrogenase (LDH)	46%
(7) Age	37%
(8) Previous biological agents	28%
(9) Lung metastases	25%
(10) Haemoglobin	22%
(11) Previous local procedures (RFA, liver resection)	3%
(12) Gender	0%
